# Pre-vaccination type-specific HPV prevalence in confirmed cervical high grade lesions in the Māori and non-Māori populations in New Zealand

**DOI:** 10.1186/s12879-015-1034-5

**Published:** 2015-08-22

**Authors:** Yoon-Jung Kang, Hazel Lewis, Megan A. Smith, Leonardo Simonella, Harold Neal, Collette Bromhead, Karen Canfell

**Affiliations:** Prince of Wales Clinical School, UNSW, Sydney, NSW 2052 Australia; Cancer Research Division, Cancer Council NSW, 153 Dowling Street, Woolloomooloo, NSW 2011 Australia; National Cervical Screening Programme, Ministry of Health, Wellington, New Zealand; Molecular Biology, Aotea Pathology, CMC Building 89 Courtenay Place, Wellington, New Zealand; Current address: 59 Normandale Rd, Lower Hutt, New Zealand; Current address: MORSE (modelling outcomes research, statistics and epidemiology) group, Roche, Basel, Switzerland; Current address: Institute of Food and Nutrition, College of Health, Massey University Wellington, Wellington, New Zealand

**Keywords:** Indigenous populations, HPV vaccine, Vaccine impact, Cervical cancer, New Zealand

## Abstract

**Background:**

New Zealand initiated HPV vaccination in 2008, and has attained 3-dose coverage of ~50 % in 12–13 year old girls. Due to the success of program initiatives in Māori girls, higher coverage rates of ~60 % have been achieved in this group. We have previously reported a benchmark overall pre-vaccination prevalence of oncogenic HPV infection in high grade cervical lesions in New Zealand. The current extended analysis provides separate pre-vaccination benchmark prevalence for Māori and non-Māori women.

**Methods:**

The National Cervical Screening Programme Register (NCSP-R) was used to identify any woman aged 20–69 years of age with an index high grade cytology report from 2009–2011. Extended recruitment was performed until 2012 in clinics with a high proportion of Māori women. Ethnicity status was based on self-reported information by participating women through phone contact supplemented by recordings on the study questionnaire (the NCSP-R was not used to extract ethnicity status). A total of 730 women consented to participate and had a valid HPV test result; 418 of these had histologically-confirmed cervical intraepithelial neoplasia (CIN) 2/3 lesions (149 Māori, 269 non-Māori). The prevalence of any cervical oncogenic HPV infection, HPV16, and HPV18 was calculated in women with CIN2/3.

**Results:**

In confirmed CIN2/3, the prevalence of any oncogenic HPV, HPV16 and HPV18 was 96 % (95 % CI:91–99 %), 54 % (95 % CI:46–63 %), 11 % (95 % CI:7–18 %) in Māori and 96 % (95 % CI:93–98 %), 54 % (95 % CI:48–60 %), 11 % (95 % CI:7–15 %) in non-Māori women, respectively. Age-specific patterns of infection for HPV16/18 in confirmed CIN2/3 differed between the two groups (P_interaction_ = 0.02), with a lower prevalence in younger *vs.* older Māori women (57 % in 20–29 years vs 75 % in 40–69 years) but a higher prevalence in younger *vs.* older non-Māori women (70 % in 20–29 years vs 49 % in 40–69 years); the difference in the age-specific patterns of infection for HPV16/18 was not significant either when considering confirmed CIN2 alone (*p* = 0.09) or CIN3 alone (*p* = 0.22).

**Conclusions:**

The overall prevalence of vaccine-included types in CIN2/3 was similar in Māori and non-Māori women, implying that the long-term effects of vaccination will be similar in the two groups.

**Electronic supplementary material:**

The online version of this article (doi:10.1186/s12879-015-1034-5) contains supplementary material, which is available to authorized users.

## Background

New Zealand initiated a national Human Papillomavirus (HPV) Immunisation Programme in 2008, which involved routine vaccination of girls aged 12–13 years from 2009 using the quadrivalent HPV6/11/16/18 vaccine, administered through a predominantly school-based immunisation program. Vaccination is also available to girls and young women up to their 18^th^ birthday through family doctors, local health centres and some Family Planning clinics [1, 2]. The reported overall population 3-dose coverage rate for girls aged 12–13 years has been stable at ~50 % since the immunisation program was introduced. However, a higher coverage rate has been achieved in Māori compared to non-Māori and non-Pacific girls (60 vs 50 % as of February 2014, in girls born in 2000) [3]: this was due to the success of specific program initiatives, which included engagement with Māori and Pacific stakeholders nationally and regionally, the involvement of Māori and Pacific Equity Advisory Groups to guide the design and roll out of the immunisation program at the regional level, and the use of the existing evidence-base to identify service delivery processes most effective for Māori and Pacific young women [4].

In New Zealand, Māori women currently experience double the rates of cervical cancer incidence and mortality compared to non-Māori women - in 2010, the age-standardised cervical cancer incidence and mortality rates in Māori women were 12.3 and 3.2 per 100,000 women, respectively, whereas the corresponding rates in non-Māori women were 6.3 and 1.6 per 100,000 women, respectively [5]. A major factor likely to underlie this difference in cancer rates is screening behaviour, and although recent screening program initiatives have been ‘closing the gap’; recent (2012) 3-yearly screening coverage rates for women aged 25–69 years in Māori and non-Māori women have been reported as 61.6 and 83.5 %, respectively [6].

A baseline measure of HPV infection in high grade cervical lesions will provide an important additional measure of the burden of high grade precancerous disease which can be substantially reduced *via* HPV vaccination in Māori women. We have previously reported the overall population prevalence of pre-vaccination oncogenic HPV infection in New Zealand in both high grade cytology and histologically-confirmed high grade lesions (cervical intraepithelial neoplasia [CIN] 2/3), in a sample of women identified *via* the New Zealand National Cervical Screening Programme (NCSP) [7]. In the current study we present findings from an updated analysis, including results from a new sample of Māori women recruited to the study. The overall aims of the current study were: (1) to compare pre-vaccination type-specific HPV prevalence in high grade disease in Māori and non-Māori women (including rates in all women with high grade cytology and in the subset of these that were histologically-confirmed CIN2/3 lesions), and (2) to assess whether the prevalence differed by age group and HPV type in CIN2/3 lesions in Māori and non- Māori women.

## Methods

### Study sample and recruitment

The initial phase of the study recruited women with a high grade cytology report occurring between August 2009 and February 2011. For the current extended analysis, the recruitment period was continued to June 2012, with the extended recruitment period focusing on recruiting Māori women only. As such, Māori women were oversampled in the final study population in order to increase the precision of estimates of HPV prevalence by ethnicity. The overall response rate to participate to the study through phone contact was 63 % (response rate by ethnicity could not be calculated since the ethnicity information came from the questionnaire). The study recruitment processes, HPV sample collection [using either SurePath^TM^ (Becton Dickinson) or ThinPrep^TM^ (Hologic) liquid based cytology (LBC)] and processing and genotyping procedures have previously been described [7]. Briefly, the New Zealand National Cervical Screening Programme Register (NCSP-R) was used to identify women aged 20–69 years of age with an incident index high grade cytology report, classified according to the Bethesda 2001 New Zealand Modified Cytology Classification System (2005) as a cytology prediction of a high grade squamous lesion (HSIL), atypical squamous cells where HSIL cannot be excluded (ASC-H), abnormal glandular cells (AGC), adenocarcinoma *in situ* (AIS), or cytology suggestive of invasion [8]. The data used from the NCSP-R related to cytology, histology, HPV testing, colposcopy exams, the National Health Index number and date of birth, but not ethnicity status.

A total of 750 women consented to participate in the study, but the final analysis was restricted to 730 women aged 20–69 years at the time of their index high grade cytology test who had a valid HPV genotyping test result. Of these, 592 (81 %) had been included in the previous analysis, which did not report results by ethnicity (we excluded two cases of 594 in a prior analysis: one had a duplicate record and the other did not have a verified HPV test record; given the very small numbers, this has no substantial impact on the prior findings) [7]. For the current analysis, women were classified as Māori or non-Māori based on self-reported information by participating women through phone contact supplemented by recordings on the study questionnaire (the NCSP-R was not used to extract ethnicity status). Women reporting multiple ethnicities of which one was Māori, were categorised as Māori women, consistent with classification used by the New Zealand Ministry of Health [9]. All other women, including women with unknown ethnicity, were categorised as non-Māori, as in other prior analyses [10]. One-third (241 of 730) of the final sample were categorised as Māori and two-thirds (489 of 730) as non-Māori. A total of 93 % overall were positive for at least one HPV type.

### HPV genotyping

HPV genotyping was performed on the LBC sample using the Linear Array genotyping system with PGMY 09/11 primers (Roche Molecular Systems, USA), using previously described methods [7, 11]. A total of 37 types of HPV were assessed (6, 11, 16, 18, 26, 31, 33, 35, 39, 40, 42, 45, 51, 52, 53, 54, 55, 56, 58, 59, 61, 62, 64, 66, 67, 68, 69, 70, 71, 72, 73, 81, 82, 83, 84, IS39 and CP6108) [12]. Due to concerns about cross-reactivity and in accordance with previous studies [7, 11], samples were considered positive for HPV type 52 only if they were also negative for HPV types 33, 35 and 58. HPV types were categorised for analysis according to the latest classification in relation to carcinogenicity as assessed by the International Agency for Research on Cancer (IARC) [13]; ‘oncogenic HPVs’ included 16, 18, 31, 33, 35, 39, 45, 51, 52, 56, 58, 59 (group 1 carcinogens) and 68 (a group 2A carcinogen).

### Histological outcomes

For the current analysis, the histological outcomes were classified according to SNOMED (1986/93) morphology categories and based on the most severe disease ranking reported after the index high grade cytology: in our data, some women had multiple histological examinations after the index high grade cytology, mostly within six month from the index cytology test. For these women, we used the most severe disease ranking in the SNOMED diagnostic (morphology M) category observed to classify histology grade. A proportion of women had histology initially recorded as ‘CIN2/3’ (M67017) as per an older SNOMED category that did not differentiate CIN2 from CIN3. For these cases, we adopted the same convention as in the NCSP-R, which was to classify these cases as CIN2; a total of 116 out of 229 CIN2 cases were originally recorded as ‘CIN2/3’.

### Statistical analysis

Comparisons between Māori and non-Māori women were made in all women with a high grade cytology report and for histologically-confirmed CIN2/3 lesions with respect to: 1) the type-specific prevalence of oncogenic HPV infection for each type; and 2) the age-specific prevalence of grouped HPV types (HPV 16, HPV 16/18, other oncogenic types).

The overall type-specific prevalence (and 95 % confidence intervals) of oncogenic HPV infection was described in all women with a high grade cytology report, as well as the subgroup of women with CIN2/3, and differences between Māori and non-Māori women were assessed using the chi-squared test. The various HPV type groupings used for analysis included: i) each specific type of oncogenic HPV infection (regardless of co-infection with other HPV types); ii) HPV 16 and/or 18, with or without co-infection with other oncogenic HPV types; iii) pooled other (non-HPV 16/18) oncogenic HPV types without co-infection with HPV 16/18; iv) pooled oncogenic HPV, either single type infection only or co-infections; and v) any HPV positive, including both oncogenic and low risk (LR) HPV types.

Women were grouped into one of three age categories (20–29, 30–39 and 40–69 years) and the age-specific prevalence of oncogenic HPV types in CIN2/3 lesions was described. Oncogenic HPV types were grouped using a hierarchical process into: i) HPV 16 positive (regardless of co-infection with other oncogenic HPV types); ii) HPV 16 and/or 18 (regardless of co-infection with other oncogenic HPV types); and iii) other oncogenic HPV types (excluding co-infection with HPV 16 and/or 18). For vaccine-included types, we described age-specific infection separately for HPV 16 and HPV 16 and/or 18.

Bivariable analyses was performed to compare the age-specific prevalence of grouped oncogenic HPV types by histology grade (CIN2 only, CIN3 only, CIN2/3) between Māori and non-Māori women, using the chi-squared test. The chi-squared test for trend across age categories was then applied, by histology grade, separately in Māori and non-Māori women. Multivariable analyses were performed to estimate the likelihood of oncogenic HPV infection, stratified by histology grade, incorporating an interaction term for ethnicity (Māori, non- Māori) and age group (20–29, 30–39 and 40–69 years). Analyses were undertaken using STATA 13 (Cary, NC, USA).

### Ethics approval

The study obtained ethics approval from the National New Zealand Ethics Committee, Cancer Council NSW Human Research Ethics Committee, Australia, and the University of Sydney Human Research Ethics Committee, Australia.

## Results

### Study sample

Clinical and demographic characteristics of the participants are described in Table 1. Of the 730 women with high grade cytology who consented to participate and had a valid HPV test result, one-third identified as Māori (241 cases). There was a slightly higher proportion of younger women (20–29 years of age) in the Māori compared to the non-Māori group (50 vs 44 %). The proportion of women with histologically-confirmed CIN2/3 lesions in Māori and non-Māori women were 62 and 55 %, respectively. A total of 96 % of confirmed CIN2+ lesions were associated with any HPV positivity.

**Table 1:** Clinical and demographic characteristics of participants (N = 730)

	Māori (N=241)	Non-Māori^a^ (N=489)	Total (N=730)	
Characteristics	No. (%)	No. (%)	No. (%)	Any HPV positive (%)
Histologically-confirmed CIN 2+	153 (63)	287 (59)	440 (60)	423 (96)
Cervical cancer^b^	4 (2)	8 (2)	12 (2)	11 (92)
High grade lesion	149 (62)	269 (55)	418 (57)	404 (97)
CIN 2^c^	80 (33)	149 (30)	229 (31)	224 (98)
CIN 3	69 (29)	120 (25)	189 (26)	180 (95)
AIS or glandular dysplasia	0 (0)	10 (2)	10 (1)	8 (80)
Histologically-confirmed < CIN 2	75 (31)	186 (38)	261 (36)	232 (89)
Low grade lesion	43 (18)	104 (21)	147 (20)	135 (92)
CIN 1	20 (8)	66 (13)	86 (12)	76 (88)
Other low grade abnormality^d^	23 (10)	38 (8)	61 (8)	59 (97)
Negative	32 (13)	82 (17)	114 (16)	97 (85)
Negative/normal	19 (8)	38 (8)	57 (8)	51 (89)
Other non-significant abnormality^e^	13 (5)	44 (9)	57 (8)	46 (81)
No biopsy taken/reported	13 (5)	16 (3)	29 (4)	24 (83)
Age 20–29 years	121 (50)	214 (44)	335 (46)	328 (98)
Age 30–39 years	77 (32)	149 (30)	226 (31)	207 (92)
Age 40–69 years	43 (18)	126 (26)	169 (23)	144 (85)

C*IN* cervical intraepithelial neoplasia, *AIS* adenocarcinoma *in situ*

^a^Women with unknown ethnicity were categorised as non-Māori

^b^Cervical cancer includes invasive adenocarcinoma, invasive squamous cell carcinoma, microinvasive squamous cell carcinoma, other primary epithelial malignancy and adenosquamous carcinoma

^c^Includes a total of 116 cases initially coded as CIN2/3 (i.e., M67017) in the National Cervical Screening Programme Register (NCSP-R); we re-classified according to the NCSP-R convention (see text)

^d^Histological appearance infection with human papillomavirus (HPV), Condyloma acuminatum, Dysplasia/CIN not otherwise specified (NOS)

^e^Inflammation, squamous metaplasia, polyp, other

### Type-specific prevalence of oncogenic HPV infection

Table 2 describes the type-specific prevalence of oncogenic HPV infection in women with high grade cytology and Table 3 describes the prevalence in women with histologically-confirmed CIN 2/3. In both groups, we identified no difference in the overall infection rates for all HPV types and for oncogenic HPV between Māori and non-Māori women. In women with high grade cytology, the infection rates in Māori vs non-Māori women for all HPV and oncogenic HPV were 95 vs 92 % (*p* = 0.14) and 92 vs 87 % (*p* = 0.08), respectively. In women with a histologically-confirmed diagnosis of CIN2/3, the corresponding rates were 96 vs 97 % (*p* = 0.57) and 96 vs 96 % (*p* = 0.37), respectively.

**Table 2:** Type-specific prevalence of oncogenic HPV infection in all women with an index high grade cytology

	High grade cytology (ASC-H/HSIL/AGC/AIS)								
	Māori (N=241)			Non-Māori (N=489)			Overall (N=730)		
HR HPV type	N	%	95 % CI	N	%	95 % CI	N	%	95 % CI
HPV16 (any)	119	49	(43–56)	208	43	(38–47)	327	45	(41–48)
HPV52 (any)	29	12	(8–17)	84	17	(14–21)	113	15	(13–18)
HPV31 (any)	33	14	(10–19)	75	15	(12–19)	108	15	(12–18)
HPV33 (any)	20	8	(5–13)	58	12	(9–15)	78	11	(9–13)
HPV18 (any)	31	13	(9–18)	54	11	(8–14)	85	12	(9–14)
HPV58 (any)	22	9	(6–13)	53	11	(8–14)	75	10	(8–13)
HPV51 (any)	21	9	(6–13)	43	9	(6–12)	64	9	(7–11)
HPV39 (any)	16	7	(4–11)	33	7	(5–9)	49	7	(5–9)
HPV45 (any)	9	4	(2–7)	24	5	(3–7)	33	5	(3–6)
HPV59 (any)	12	5	(3–9)	23	5	(3–7)	35	5	(3–7)
HPV35 (any)	9	4	(2–7)	18	4	(2–6)	27	4	(3–5)
HPV56 (any)	12	5	(3–9)	12	3	(1–4)	24	3	(2–5)
HPV68 (any)	1	0	(0–2)	15	3	(2–5)	16	2	(1–4)
HPV16 and/or 18 (any)	141	59	(52–65)	252	52	(47–56)	393	54	(50–57)
HPV16 with HPV18 (any)	9	4	(2–7)	9	2	(1–4)	18	3	(2–4)
HPV16 without HPV18 (any)	110	46	(39–52)	199	41	(36–45)	309	42	(39–46)
HPV18 without HPV16 (any)	22	9	(6–13)	44	9	(7–12)	66	9	(7–11)
HPV16 and/or 18 (without OHR)	88	37	(30–43)	115	24	(20–28)	203	28	(25–31)
HPV16 and/or 18 (with OHR)	53	22	(17–28)	137	28	(24–32)	190	26	(23–29)
OHR without HPV 16/18	82	34	(28–40)	171	35	(31–39)	253	35	(31–38)
HR HPV (single infection)	137	57	(50–63)	212	43	(39–48)	349	48	(44–52)
HR HPV (any)	222	92	(88–95)	423	87	(83–89)	645	88	(86–91)
HPV positive (including LR HPV)	229	95	(91–97)	450	92	(89–94)	679	93	(91–95)

*HR* high risk, *LR* low risk, *OHR* Other high risk, excluding HPV 16 or 18; any – regardless of co-infection with other HPV types

HR HPV includes infection with either type 16, 18, 31, 33, 35, 39, 45, 51, 52, 56, 58, 59 or 68

P values for differences were not calculated for each specific HPV type between Māori and non-Māori women due to sample size limitations and concerns about multiple comparisons

**Table 3:** Type-specific prevalence of oncogenic HPV infection in women with histologically-confirmed CIN 2/3

	CIN2/3 combined (N = 418)								
	Māori (N=149)			Non-Māori (N=269)			Overall (N=418)		
HR HPV type	N	%	95 % CI	N	%	95 % CI	N	%	95 % CI
HPV16 (any)	81	54	(46–63)	146	54	(48–60)	227	54	(49–59)
HPV52 (any)	22	15	(10–21)	53	20	(15–25)	75	18	(14–22)
HPV31 (any)	19	13	(8–19)	52	19	(15–25)	71	17	(14–21)
HPV33 (any)	15	10	(6–16)	38	14	(10–19)	53	13	(10–16)
HPV18 (any)	17	11	(7–18)	29	11	(7–15)	46	11	(8–14)
HPV58 (any)	16	11	(6–17)	36	13	(10–18)	52	12	(9–16)
HPV51 (any)	15	10	(6–16)	26	10	(6–14)	41	10	(7–13)
HPV39 (any)	12	8	(4–14)	19	7	(4–11)	31	7	(5–10)
HPV45 (any)	6	4	(2–9)	13	5	(3–8)	19	5	(3–7)
HPV59 (any)	9	6	(3–11)	13	5	(3–8)	22	5	(3–8)
HPV35 (any)	6	4	(2–9)	11	4	(2–7)	17	4	(2–6)
HPV56 (any)	8	5	(2–10)	4	2	(0–4)	12	3	(2–5)
HPV68 (any)	1	1	(0–4)	9	3	(2–6)	10	2	(1–4)
HPV16 and/or 18 (any)	92	62	(53–70)	167	62	(56–68)	259	62	(57–67)
HPV16 with HPV18 (any)	6	4	(2–9)	7	3	(1–5)	13	3	(2–5)
HPV16 without HPV18 (any)	75	50	(42–59)	139	52	(46–58)	214	51	(46–56)
HPV18 without HPV16 (any)	11	7	(4–13)	21	8	(5–12)	32	8	(5–11)
HPV16 and/or 18 (without OHR)	56	38	(30–46)	72	27	(22–32)	128	31	(26–35)
HPV16 and/or 18 (with OHR)	36	24	(18–32)	95	35	(30–41)	131	31	(27–36)
OHR without HPV 16/18	51	34	(27–42)	92	34	(29–40)	143	34	(30–39)
HR HPV (single infection)	85	57	(49–65)	123	46	(40–52)	208	50	(45–55)
HR HPV (any)	143	96	(91–99)	258	96	(93–98)	401	96	(94–98)
HPV positive (including LR HPV)	143	96	(91–99)	261	97	(94–99)	404	97	(94–98)

*HR* high risk, *LR* low risk, *OHR* Other high risk, excluding HPV 16 or 18; any – regardless of co-infection with other HPV types

HR HPV includes infection with either type 16, 18, 31, 33, 35, 39, 45, 51, 52, 56, 58, 59 or 68

P values for differences were not calculated for each specific HPV type between Māori and non-Māori women due to sample size limitations and concerns about multiple comparisons

In general, the type-specific prevalence of each oncogenic HPV infection appeared broadly similar in Māori and non-Māori women either when considering all women with high grade cytology or when the group with histologically confirmed CIN2/3 were considered. The most common HPV types in women with high grade cytology in the study (Māori vs non-Māori women) were HPV 16 (49 vs 43 %), HPV 52 (12 vs 17 %), HPV 31 (14 vs 15 %), HPV 33 (8 vs 12 %) and HPV 18 (13 vs 11 %). Similarly, the most common HPV types in CIN 2/3 lesions (Māori vs non-Māori women) were HPV 16 (54 vs 54 %), HPV 52 (15 vs 20 %), HPV 31 (13 vs 19 %), HPV 33 (10 vs 14 %) and HPV 18 (11 vs 11 %).

The prevalence of HPV 16 and/or 18, regardless of co-infection with other oncogenic HPV types, did not significantly differ between Māori and non-Māori women either with high grade cytology (59 vs 52 %, *p* = 0.12) or with histologically-confirmed high grade disease (62 vs 62 %, *p* = 0.85). Similarly, the prevalence of other oncogenic HPV types without co-infection with HPV 16/18 was similar between Māori and non-Māori women either with high grade cytology (34 vs 35 %, p = 0.28) or with histologically-confirmed high grade disease (34 vs 34 %, p = 0.85). The overall prevalence of HPV 16 and/or 18 without co-infection with other oncogenic HPV types was significantly higher in Māori than non-Māori women either with high grade cytology (37 vs 24 %, *p* = 0.001) or with histologically-confirmed high grade disease (38 vs 27 %, *p* = 0.05).

Further details on the type-specific prevalence of oncogenic HPV infection in women with CIN2 only, CIN3 only, CIN2+ (including AIS or glandular dysplasia and cancer) and CIN3+ (including AIS or glandular dysplasia and cancer) are provided in Supplementary data (Additional file 1: Tables S1–S4).

### Age-specific prevalence of oncogenic HPV infection

**Fig. 1:**
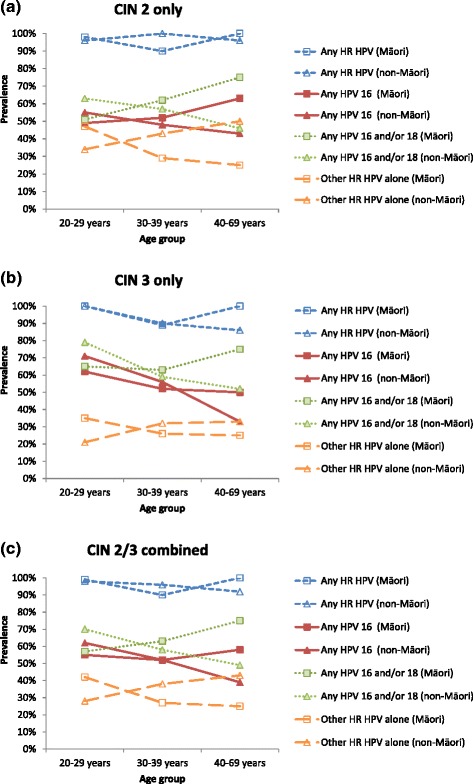
Age-specific prevalence of oncogenic HPV types by histology grade and ethnicity. Any HR HPV – any high risk (HR) HPV infection, regardless of co-infection with other HPV types; Any HPV 16 - HPV 16 infection, regardless of co-infection with other oncogenic HPV types; Any HPV 16 and/or 18 - HPV 16 and/or 18 infection, regardless of co-infection with other oncogenic HPV types; Other HR HPV alone - other high risk HPV infection without co-infection with HPV 16 and/or 18

The age-specific prevalence of any oncogenic HPV infection in women with histologically-confirmed high grade disease was not found to be significantly different between Māori and non-Māori (Fig. 1). Bivariable analyses showed that the age-specific prevalence of HPV 16 regardless of co-infection with other HPV types, HPV 16 and/or 18 regardless of co-infection with other HPV types, or other oncogenic HPV without HPV 16 and/or 18, within each histology grade (CIN 2 only, CIN 3 only, CIN 2/3) was not statistically different between Māori and non-Māori women (Table 4).

**Table 4:** Age-specific prevalence of grouped HPV types by histology grade

		CIN 2 (N = 229)					CIN 3 (N = 189)					CIN 2/3 combined (N = 418)				
		Māori		Non-Māori		X^2^ test^b^	Māori		Non-Māori		X^2^ test^b^	Māori		Non-Māori		X^2^ test^b^
HR HPV	Age group	N^a^	% (95 % CI)	N^a^	% (95 % CI)	*P* value	N^a^	% (95 % CI)	N^a^	% (95 % CI)	*P* value	N^a^	% (95 % CI)	N^a^	% (95 % CI)	*P* value
HPV 16 (any)	20–29	43	49 (33–65)	67	55 (43–67)	0.51	34	62 (44–78)	58	71 (57–82)	0.38	77	55 (43–66)	125	62 (53–71)	0.27
	30–39	21	52 (30–74)	54	48 (34–62)	0.74	27	52 (32–71)	41	56 (40–72)	0.73	48	52 (37–67)	95	52 (41–62)	0.95
	40–69	16	63 (35–85)	28	43 (24–63)	0.21	8	50 (16–84)	21	33 (15–57)	0.41	24	58 (37–78)	49	39 (25–54)	0.11
	Test for trend ^c^	*P* = 0.37		*P* = 0.26		–	*P* = 0.56		*P* = 0.01		–	*P* = 0.75		*P* = 0.01		–
HPV 16 and/or	20–29	43	51 (35–67)	67	63 (50–74)	0.49	34	65 (46–80)	58	79 (67–89)	0.12	77	57 (45–68)	125	70 (62–78)	0.16
18 (any)	30–39	21	62 (38–82)	54	57 (43–71)	0.05	27	63 (42–81)	41	59 (42–74)	0.88	48	63 (47–76)	95	58 (47–68)	0.21
	40–69	16	75 (48–93)	28	46 (28–66)	0.17	8	75 (35–97)	21	52 (30–74)	0.46	24	75 (53–90)	49	49 (34–64)	0.08
	Test for trend ^c^	*P* = 0.09		*P* = 0.17		–	*P* = 0.51		*P* = 0.05		–	*P* = 0.09		*P* = 0.02		–
OHR without	20–29	43	47 (31–62)	67	34 (23–47)	0.40	34	35 (20–54)	58	21 (11–33)	0.12	77	42 (30–53)	125	28 (20–37)	0.12
HPV 16/18	30–39	21	29 (11–52)	54	43 (29–57)	0.05	27	26 (11–46)	41	32 (18–48)	0.88	48	27 (15–42)	95	38 (28–48)	0.21
	40–69	16	25 (7–52)	28	50 (31–69)	0.17	8	25 (3–65)	21	33 (15–57)	0.55	24	25 (10–47)	49	43 (29–58)	0.11
	Test for trend ^c^	*P* = 0.09		*P* = 0.12		–	*P* = 0.51		*P* = 0.1		–	*P* = 0.09		*P* = 0.02		–

*HR* high risk, *OHR* Other high risk, excluding HPV 16 or 18; any – regardless of co-infection with other HPV types

HR HPV includes infection with either type 16, 18, 31, 33, 35, 39, 45, 51, 52, 56, 58, 59 or 68

^a^Total number of women at each age group by ethnicity (*i.e.,* including women HPV negative)

^b^Chi-square test for difference between Māori and non-Māori women in the age-specific prevalence of grouped HPV types

^c^Test for trend across each level of age group

In general, a significant trend in pooled HPV infection rates (all types) across age groups was not observed in Māori women with histologically-confirmed high grade disease, although younger Māori women tended to have a lower relative prevalence of HPV 16 and/or 18 and a higher prevalence of other oncogenic HPV types compared to older Māori women. In CIN2/3 lesions, the prevalence of HPV 16/18 in younger (20–29 years) *vs.* older (40–69 years) Māori women was 57 and 75 %, respectively; the prevalence of other oncogenic HPV types in younger vs older Māori women was 42 and 25 %, respectively. In CIN2 lesions only, the prevalence of HPV 16/18 in younger *vs.* older Māori women was 51 and 75 %, respectively; the prevalence of other oncogenic HPV types in younger vs older was 47 and 25 %, respectively. In CIN3 only, the prevalence of HPV 16/18 in younger *vs.* older Māori women was 65 and 75 %, respectively; the prevalence of other oncogenic HPV types was 35 and 25 %, respectively. The prevalence of HPV 16, regardless of co-infection with other oncogenic HPV types, in younger *vs.* older Māori women with CIN2/3, CIN2 and CIN3 was 55 vs 58 %, 49 vs 63 % and 62 vs 50 %, respectively (Table 4). In Māori women with histologically-confirmed CIN 2/3, the prevalence of single infection for any oncogenic HPV did not change substantially with age (53 % in 20–29 years vs 60 % in 40–69 years; *p* = 0.27). But as women became older, the prevalence of HPV 16 and/or 18 alone (*i.e.,* without co-infection with other oncogenic HPV types) sharply increased from 30 % (20–29 years) to 50 % (40–69 years) (*p* = 0.03), and the prevalence of other oncogenic HPV infection alone (*i.e.,* without HPV 16/18) decreased substantially from 42 % (20–29 years) to 25 % (40–69 years; *p* = 0.09) (Additional file 1: Figure S1).

By contrast, younger non-Māori women had a higher relative prevalence of HPV 16 and/or 18 and a lower prevalence of other oncogenic HPV types, compared to older non-Māori women. In CIN2/3 lesions, younger non-Māori women, compared to older non-Māori women, had a significantly higher prevalence of HPV 16 and/or 18 (70 % in 20–29 years vs 49 % in 40–69 years, *p* = 0.02); the prevalence of other oncogenic HPV types was significantly lower in younger non-Māori women (28 % in 20–29 years vs 43 % in 40–69 years, *p* = 0.02). In CIN2 lesions only, no significant trend in the prevalence of grouped oncogenic HPV by age categories was observed in non-Māori women; the prevalence of HPV 16/18 in younger *vs.* older Māori women was 63 and 46 %, respectively, and the prevalence of other oncogenic HPV types in younger vs older was 34 and 50 %, respectively. In CIN3 only, younger non-Māori women had a significantly higher prevalence of HPV 16 and/or 18 (79 % in 20–29 years vs 52 % in 40–69 years, *p* = 0.05); the prevalence of other oncogenic HPV types was not significantly different by age categories (21 % in 20–29 years vs 33 % in 40–69 years). The prevalence of HPV 16, regardless of co-infection with other oncogenic HPV types, in younger *vs.* older non-Māori women with CIN2/3, CIN2 and CIN3 was 62 vs 39 % (*p* = 0.01), 55 vs 43 % (*p* = 0.26) and 71 vs 33 % (*p* = 0.01), respectively (Table 4). In non-Māori women with histologically-confirmed CIN 2/3, the prevalence of other oncogenic HPV regardless of co-infection with HPV 16 and/or 18 was similar at each age group (~70 %). As women became older, the prevalence of co-infection between other oncogenic HPV types and HPV 16 and/or 18 decreased from 70 % (20–29 years) to 49 % (40–69 years) (*p* = 0.02) as the infection for HPV 16 and/or 18 alone became slightly less prevalent (from 29 % in 20–29 years down to 22 % in 40–69 years) (Additional file 1: Figure S1).

The apparent contrasting age-specific pattern of infection for HPV 16 and/or 18 between the two ethnic groups (*i.e.,* a lower prevalence in younger *vs.* older Māori women and a higher prevalence of younger *vs.* older non-Māori women) was significant in a multivariable analysis for CIN2/3 lesions (*p* = 0.02), but not for CIN2 alone (*p* = 0.09) or CIN3 alone (*p* = 0.22), separately. Similarly, the inverse age-specific pattern of infection for other oncogenic HPV between the two ethnic groups (*i.e.,* a relatively higher prevalence in younger *vs.* older Māori women and a relatively lower prevalence of younger *vs.* older non-Māori women) was significant for CIN2/3 lesions (*p* = 0.02), but not CIN2 alone (*p* = 0.07) or CIN3 alone (*p* = 0.27). The effect of age group on the likelihood of having infection for HPV 16 only did not significantly differ between Māori and non-Māori women in high grade lesions (Table 5).

**Table 5:** Likelihood of having grouped HPV infection in HPV positive women with histologically confirmed high grade lesion at each level of age group by ethnicity

Category	Any HPV 16 positive^a^			Any HPV 16 and/or 18 positive^a^			Other HR HPV without HPV 16 and/or 18		
	CIN 2	CIN 3	CIN 2/3	CIN 2	CIN 3	CIN 2/3	CIN 2	CIN 3	CIN 2/3
	OR (95 % CI)	OR (95 % CI)	OR (95 % CI)	OR (95 % CI)	OR (95 % CI)	OR (95 % CI)	OR (95 % CI)	OR (95 % CI)	OR (95 % CI)
Non-Māori, 20–29 years	1.0	1.0	1.0	1.0	1.0	1.0	1.0	1.0	1.0
Non-Māori, 30–39 years	0.8 (0.4–1.5)	0.5 (0.2–1.2)	0.6 (0.4–1.1)	0.8 (0.4–1.6)	0.5 (0.2–1.2)	0.6 (0.4–1.1)	1.4 (0.7–2.9)	2.1 (0.8–5.2)	1.7 (0.9–3.0)
Non-Māori, 40–69 years	0.6 (0.3–1.5)	0.2 (0.1–0.6)	0.4 (0.2–0.8)	0.5 (0.2–1.3)	0.4 (0.1–1.1)	0.4 (0.2–0.9)	2.0 (0.8–5.0)	2.2 (0.7–6.9)	2.1 (1.1–4.3)
Māori, 20–29 years	0.8 (0.4–1.7)	0.7 (0.3–1.6)	0.7 (0.4–1.3)	0.6 (0.3–1.4)	0.5 (0.2–1.2)	0.6 (0.3–1.0)	1.7 (0.8–3.7)	2.1 (0.8–5.4)	1.8 (1.0–3.4)
Māori, 30–39 years	0.9 (0.3–2.4)	0.4 (0.2–1.1)	0.7 (0.3–1.3)	1.2 (0.4–3.7)	0.6 (0.2–1.9)	0.9 (0.4–2.0)	0.9 (0.3–2.6)	1.6 (0.5–4.7)	1.1 (0.5–2.4)
Māori, 40–69 years	1.4 (0.4–4.1)	0.4 (0.1–1.9)	0.8 (0.3–2.1)	1.7 (0.5–5.9)	0.8 (0.1–4.4)	1.2 (0.5–3.3)	0.6 (0.2–2.2)	1.3 (0.2–7.1)	0.8 (0.3–2.3)
Test for interaction^b^	*P* = 0.36	*P* = 0.52	*P* = 0.16	*P* = 0.09	*P* = 0.22	*P* = 0.02	*P* = 0.07	*P* = 0.27	*P* = 0.02

Other HR HPV types include infection with either type 31, 33, 35, 39, 45, 51, 52, 56, 58, 59 or 68, not 16 or 18

^a^Regardless of co-infection with other HPV types

^b^Logistic regression including an interaction term for ethnicity and age group

Further details on the age-specific prevalence of grouped oncogenic HPV infection in women with high grade cytology, CIN2+ (including AIS or glandular dysplasia and cancer) and CIN3+ (including AIS or glandular dysplasia and cancer) are provided in Additional file 1: Figure S2, Table S5 and Table S6.

## Discussion

This is the first study to estimate the type-specific prevalence of oncogenic HPV infection according to ethnicity in New Zealand, and it also represents one of the most extensive analyses in any setting of the prevalence of oncogenic HPV infection in histologically-confirmed high grade lesions. The study was conducted at the onset of the HPV Immunisation Programme in New Zealand, and thus provides a benchmark for pre-vaccination type-specific HPV prevalence rates in high grade lesions in Māori and non-Māori women. We found no difference in the overall rate of oncogenic HPV infections, or the rate of HPV 16/18 infections, between Māori and non-Māori women in high grade disease. We found that HPV 16 and/or 18 was present in 62 % of histologically-confirmed CIN2/3 lesions overall, for both Māori and non-Māori women. However we did identify a possible contrasting age-specific pattern of infection for HPV 16/18 in confirmed CIN2/3 lesions between the two ethnic groups, such that there was a relatively lower prevalence in younger *vs.* older Māori women and a higher prevalence in younger *vs.* older non-Māori women; although this difference was not borne out when CIN2 and CIN3 were considered separately.

Although our findings for an increased relative prevalence of HPV16/18 in CIN2/3 from younger, compared to those from older, non-Māori women is broadly consistent with prior findings from a cohort in Guanacaste, Costa Rica [14], the interaction identified in the current study between the age-specific relative prevalence and ethnicity has not previously been reported. However, although we found a significant interaction between age and ethnicity in predicting the relative prevalence of HPV16/18 in CIN 2/3, this pattern for a significantly different, age-specific effect was not confirmed when only CIN3 lesions, the most serious grade of precancer, were compared between ethnic groups. Taken together therefore, our findings imply that HPV vaccination with current generation vaccines is expected to have a similar impact in Māori and non-Māori women in the longer term. However, there is an ongoing need to prioritise cervical screening initiatives in Māori as well as other groups of women in New Zealand (for example Pacific women), who have historically lower screening participation rates [6].

Our study has some limitations. It is possible that women who consented to participate in the study might differ from those who did not in terms of demographic characteristics or cervical screening history, which may have impacted the type-specific rates of HPV in high grade lesions, and that this difference might vary between ethnic groups. However, in our previous analysis we showed that the sample of women consenting to the study was representative of those with high grade lesions in the New Zealand NCSP Register [6]. It is possible that significant differences between the prevalence of specific HPV types exist between Māori and non-Māori women; we were not able to test for this due to sample size limitations and concerns about multiple comparisons. Therefore, we have focused here mainly on our findings for the main groups of relevant HPV types (*i.e.,* HPV 16/18 [the vaccine included oncogenic types] and all other oncogenic types considered as a group). It is also possible that we did not detect an interaction between age and ethnicity for specific lesion grades because of a lack of power to detect such an interaction. Note also that we did not control for deprivation or other factors potentially underlying our findings for Māori vs. non-Māori women. The primary aim of the study was to describe potential differences in type-specific HPV prevalence by ethnicity, rather than explore mechanisms that underlie ethnic differences in the prevalence. The importance of doing so is that in New Zealand targeted programs are implemented for specific ethnic groups (irrespective of deprivation). We have therefore configured the analysis to be directly relevant to this approach.

In line with our previous analysis [7], the very high (96 %) overall oncogenic HPV infection rate we observed in confirmed CIN2/3 lesions appears to be somewhat higher than reported in other studies and regions; possible reasons include improvements in PCR technology used to detect and genotype HPV infection [12] as well as our concentrated recruitment of an ‘enriched’ population of women who had both high grade cytology and a subsequently confirmed high grade CIN (by contrast, many prior studies of HPV prevalence in CIN2/3 have tested samples identified *via* laboratory reports of CIN2/3 for which biopsy may have been originally performed for a range of indications) [15]. Despite the use of an enriched population in our study, the relative prevalence of specific HPV types in CIN2/3 in the current study is broadly consistent with results from a recent meta-analysis of international studies [16]. The most common HPV types detected in CIN2/3 lesions in the current analysis and meta-analysis of other international studies were, respectively, HPV 16 (54 vs 53 %), HPV 52 (18 vs 12 %), HPV 31 (17 vs 12 %), HPV 33 (13 vs 9 %) and HPV 18 (11 vs 8 %) [16].

It is known that type-specific prevalence of HPV varies substantially by geographical regions [16, 17]. However, globally, relatively little data are available on comparative type-specific HPV prevalence by ethnicity when a sample of women are drawn from the same geographical area over the same period, especially in women with high grade lesions. We identified one recent US study, which found a significant difference (p < 0.0001) in the prevalence of HPV 16/18 by ethnicity (with or without co-infection with other oncogenic HPV types) in women 18–39 years of age with CIN2+, after adjusting for age, diagnosis grade and geographic location [18]. The prevalence of HPV 16/18 (regardless of co-infection with other oncogenic HPV types) was the highest in Non-Hispanic whites (59.1 %) followed by Hispanic (46.3 %), Asian (43.3 %) and Non-Hispanic black (41.9 %) women. The adjusted prevalence ratio (APR) of having HPV 16/18 vs other oncogenic HPV types in CIN2+ was significantly lower in Non-Hispanic black (APR = 0.70, 95 % CI: 0.62–0.80) and Hispanic women (APR = 0.83, 95 % CI: 0.74–0.93), compared to Non-Hispanic white women, and the unadjusted prevalence ratio was similar to the APR [18]. Therefore, the US study identifies the potential for HPV vaccine impact to vary by ethnic group (in a way that is independent from, and additional to, the effect of potentially differing coverage between groups).

Although the results of general population HPV prevalence studies are not directly comparable with our findings, we identified two such studies assessing type-specific prevalence of HPV by ethnicity or Indigenous status. In Canada, HPV prevalence was assessed among women with aboriginal, Caucasian and other ethnic backgrounds (81 % of participants were women with normal cytology) [19]. The study found that overall HPV prevalence in women <30 years was similar in Aboriginal and Caucasian women, but in women aged over 30 years, HPV prevalence was significantly higher in Aboriginal women. In Australia, type-specific population prevalence was assessed in Indigenous and non-Indigenous women 15–40 years of age attending for a routine Pap smear in the WHINURS study [20]. It was found that Indigenous and non-Indigenous women 30–40 years of age had a similar infection rate for HPV 16/18, but Indigenous women had a significantly higher infection for other oncogenic HPV types (35 vs 22.5 %, p < 0.001). In women aged 15–29 years, Indigenous and non-Indigenous women had similar infection rates for HPV 16/18 and for other oncogenic HPV types. In this study, a lower prevalence of HPV type 68 was reported in Indigenous women (0.9 vs 3.2 %, p < 0.001), but the authors stated that this finding need to be interpreted with caution given that multiple comparisons of genotype groups were performed. In general terms, although these studies provide some basis for concluding that HPV type-specific infection rates can potentially differ between ethnic groups in the same population, general population studies are likely to be more limited in their power to detect differences between groups because of the lower absolute prevalence of HPV in the general population compared to the group with high grade lesions.

## Conclusion

Our study, therefore, represents one of the first detailed investigations of the relative prevalence of vaccine-included HPV types which can directly compare findings across ethnic groups. Our finding that the overall prevalence of vaccine-included HPV types in CIN2/3 is similar in Māori and non-Māori women is reassuring, and implies that the longer term effects of vaccination will be similar in the two groups, or even (given the higher coverage rates achieved), that better post-vaccination outcomes might be achieved in Māori groups. We conclude that ongoing high coverage of both Māori and non-Māori women in the National HPV Immunisation Programme in New Zealand is required to achieve high overall program effectiveness, and our results also underpin the ongoing importance of initiatives to further increase participation in cervical screening in Māori women. In the future, repeat cross-sectional surveys of the type reported here will provide an ongoing mechanism of monitoring the impact of HPV vaccination in New Zealand. To facilitate ongoing monitoring of the impact of HPV vaccination on the National Cervical Screening Program, individual record linkage between the NCSP-R and immunisation registries is under active discussion.

## Additional file

Additional file 1: Figure S1. Age-specific prevalence of grouped oncogenic HPV types in CIN 2/3 lesions in (a) Māori women and (b) non-Māori women. **Figure S2.** Age-specific prevalence of grouped oncogenic HPV types by histology grade in women with: (a) high grade cytology; (b) histologically-confirmed CIN 2+; and (c) histologically-confirmed CIN 3+, including AIS or glandular dysplasia and cancer, by ethnicity. **Table S1.** Type-specific prevalence of oncogenic HPV infection in women with histologically-confirmed CIN 2 only. **Table S2.** Type-specific prevalence of oncogenic HPV infection in women with histologically-confirmed CIN 3 only. **Table S3.** Type-specific prevalence of oncogenic HPV infection in women with histologically-confirmed CIN2+. **Table S4.** Type-specific prevalence of oncogenic HPV infection in women with histologically-confirmed CIN3+. **Table S5.** Age-specific prevalence of grouped HPV types in women with (a) high grade cytology; (b) histologically-confirmed CIN 2+; and (c) histologically-confirmed CIN 3+, including AIS or glandular dysplasia and cancer. **Table S6.** Likelihood of having grouped HPV infection in HPV positive women with (a) high grade cytology; (b) histologically-confirmed CIN 2+ (including cancer); and (c) histologically-confirmed CIN 3+ (including cancer). (DOCX 202 kb)

## Abbreviations

HPV: Human papillomavirus
CIN: Cervical intraepithelial neoplasia
NCSP-R: National Cervical Screening Programme Register
CI: Confidence interval
