# Supplementary material for: Pre-vaccination type-specific HPV prevalence in confirmed cervical high grade lesions in the Māori and non-Māori populations in New Zealand
Source: BMC Infect Dis. 2015 Aug 22;15:365. doi: 10.1186/s12879-015-1034-5 (PMC4546322; doi:10.1186/s12879-015-1034-5)
Supplement: Additional file 1: Figure S1. — Age-specific prevalence of grouped oncogenic HPV types in CIN 2/3 lesions in (a) Māori women and (b) non-Māori women. Figure S2. Age-specific prevalence of grouped oncogenic HPV types by histology grade in women with: (a) high grade cytology; (b) histologically-confirmed CIN 2+; and (c) histologically-confirmed CIN 3+, including AIS or glandular dysplasia and cancer, by ethnicity. Table S1. Type-specific prevalence of oncogenic HPV infection in women with histologically-confirmed CIN 2 only. Table S2. Type-specific prevalence of oncogenic HPV infection in women with histologically-confirmed CIN 3 only. Table S3. Type-specific prevalence of oncogenic HPV infection in women with histologically-confirmed CIN2+. Table S4. Type-specific prevalence of oncogenic HPV infection in women with histologically-confirmed CIN3+. Table S5. Age-specific prevalence of grouped HPV types in women with (a) high grade cytology; (b) histologically-confirmed CIN 2+; and (c) histologically-confirmed CIN 3+, including AIS or glandular dysplasia and cancer. Table S6. Likelihood of having grouped HPV infection in HPV positive women with (a) high grade cytology; (b) histologically-confirmed CIN 2+ (including cancer); and (c) histologically-confirmed CIN 3+ (including cancer). (DOCX 202 kb) [file 12879_2015_1034_MOESM1_ESM.docx]

Figure 1A. Age-specific prevalence of grouped oncogenic HPV types in CIN 2/3 lesions in (a) Māori women and (b) non-Māori women

Figure 2A. Age-specific prevalence of grouped oncogenic HPV types by histology grade in women with: (a) high grade cytology; (b) histologically-confirmed CIN 2+; and (c) histologically-confirmed CIN 3+, including AIS or glandular dysplasia and cancer, by ethnicity

Any HR HPV – any high risk (HR) HPV infection, regardless of co-infection with other HPV types; Any HPV 16 - HPV 16 infection, regardless of co-infection with other oncogenic HPV types; Any HPV 16 and/or 18 - HPV 16 and/or 18 infection, regardless of co-infection with other oncogenic HPV types; Other HR HPV alone - other high risk HPV infection without co-infection with HPV 16 and/or 18.

Table 1A. Type-specific prevalence of oncogenic HPV infection in women with histologically-confirmed CIN 2 only

|  | **CIN 2 only (N=229)** | | | | | | | | |
| --- | --- | --- | --- | --- | --- | --- | --- | --- | --- |
|  | **Māori** | | | **Non-Māori** | | | **Overall** | | |
| **HR HPV type** | **N** | **%** | **95% CI** | **N** | **%** | **95% CI** | **N** | **%** | **95% CI** |
| **HPV16 (any)** | **42** | **52** | **(41-64)** | **75** | **50** | **(42-59)** | **117** | **51** | **(44-58)** |
| HPV52 (any) | 10 | 13 | (6-22) | 26 | 17 | (12-25) | 36 | 16 | (11-21) |
| HPV31 (any) | 12 | 15 | (8-25) | 36 | 24 | (18-32) | 48 | 21 | (16-27) |
| HPV33 (any) | 10 | 13 | (6-22) | 26 | 17 | (12-25) | 36 | 16 | (11-21) |
| HPV18 (any) | 6 | 8 | (3-16) | 16 | 11 | (6-17) | 22 | 10 | (6-14) |
| HPV58 (any) | 10 | 13 | (6-22) | 23 | 15 | (10-22) | 33 | 14 | (10-20) |
| HPV51 (any) | 7 | 9 | (4-17) | 17 | 11 | (7-18) | 24 | 10 | (7-15) |
| HPV39 (any) | 6 | 8 | (3-16) | 10 | 7 | (3-12) | 16 | 7 | (4-11) |
| HPV45 (any) | 1 | 1 | (0-7) | 6 | 4 | (2-9) | 7 | 3 | (1-6) |
| HPV59 (any) | 5 | 6 | (2-14) | 8 | 5 | (2-10) | 13 | 6 | (3-10) |
| HPV35 (any) | 6 | 8 | (3-16) | 8 | 5 | (2-10) | 14 | 6 | (3-10) |
| HPV56 (any) | 4 | 5 | (1-12) | 3 | 2 | (0-6) | 7 | 3 | (1-6) |
| HPV68 (any) | 1 | 1 | (0-7) | 5 | 3 | (1-8) | 6 | 3 | (1-6) |
| **HPV16 and/or 18 (any)** | **47** | **59** | **(47-70)** | **86** | **58** | **(49-66)** | **133** | **58** | **(51-65)** |
| HPV16 with HPV18 (any) | 1 | 1 | (0-7) | 5 | 3 | (1-8) | 6 | 3 | (1-6) |
| HPV16 without HPV18 (any) | 41 | 51 | (40-63) | 70 | 47 | (39-55) | 111 | 48 | (42-55) |
| HPV18 without HPV16 (any) | 5 | 6 | (2-14) | 11 | 7 | (4-13) | 16 | 7 | (4-11) |
| HPV16 and/or 18 (without OHR) | 29 | 36 | (26-48) | 32 | 21 | (15-29) | 61 | 27 | (21-33) |
| HPV16 and/or 18 (with OHR) | 18 | 22 | (14-33) | 54 | 36 | (29-45) | 72 | 31 | (25-38) |
| **OHR without HPV 16/18** | **30** | **38** | **(27-49)** | **60** | **40** | **(32-49)** | **90** | **39** | **(33-46)** |
| HR HPV (single infection) | 44 | 55 | (43-66) | 64 | 43 | (35-51) | 108 | 47 | (41-54) |
| **HR HPV (any)** | **77** | **96** | **(89-99)** | **145** | **97** | **(93-99)** | **222** | **97** | **(94-99)** |
| **HPV positive (including LR HPV)** | **77** | **96** | **(89-99)** | **147** | **99** | **(95-100)** | **224** | **98** | **(95-99)** |

HR – high risk; LR – low risk; OHR – Other high risk, excluding HPV 16 or 18; any – regardless of co-infection with other HPV types.

HR HPV includes infection with either type 16, 18, 31, 33, 35, 39, 45, 51, 52, 56, 58, 59 or 68.

P values for differences were not calculated for each specific HPV type between Māori and non-Māori women due to sample size limitations and concerns about multiple comparisons.

Table 2A. Type-specific prevalence of oncogenic HPV infection in women with histologically-confirmed CIN 3 only

|  | **CIN 3 only (N=189)** | | | | | | | | |
| --- | --- | --- | --- | --- | --- | --- | --- | --- | --- |
|  | **Māori** | | | **Non-Māori** | | | **Overall** | | |
| **HR HPV type** | **N** | **%** | **95% CI** | **N** | **%** | **95% CI** | **N** | **%** | **95% CI** |
| **HPV16 (any)** | **39** | **57** | **(44-68)** | **71** | **59** | **(50-68)** | **110** | **58** | **(51-65)** |
| HPV52 (any) | 12 | 17 | (9-28) | 27 | 22 | (15-31) | 39 | 21 | (15-27) |
| HPV31 (any) | 7 | 10 | (4-20) | 16 | 13 | (8-21) | 23 | 12 | (8-18) |
| HPV33 (any) | 5 | 7 | (2-16) | 12 | 10 | (5-17) | 17 | 9 | (5-14) |
| HPV18 (any) | 11 | 16 | (8-27) | 13 | 11 | (6-18) | 24 | 13 | (8-18) |
| HPV58 (any) | 6 | 9 | (3-18) | 13 | 11 | (6-18) | 19 | 10 | (6-15) |
| HPV51 (any) | 8 | 12 | (5-22) | 9 | 8 | (4-14) | 17 | 9 | (5-14) |
| HPV39 (any) | 6 | 9 | (3-18) | 9 | 8 | (4-14) | 15 | 8 | (5-13) |
| HPV45 (any) | 5 | 7 | (2-16) | 7 | 6 | (2-12) | 12 | 6 | (3-11) |
| HPV59 (any) | 4 | 6 | (2-14) | 5 | 4 | (1-10) | 9 | 5 | (2-9) |
| HPV35 (any) | 0 | 0 | (0-5) | 3 | 3 | (1-7) | 3 | 2 | (0-5) |
| HPV56 (any) | 4 | 6 | (2-14) | 1 | 1 | (0-5) | 5 | 3 | (1-6) |
| HPV68 (any) | 0 | 0 | (0-5) | 4 | 3 | (1-8) | 4 | 2 | (1-5) |
| **HPV16 and/or 18 (any)** | **45** | **65** | **(53-76)** | **81** | **68** | **(58-76)** | **126** | **67** | **(59-73)** |
| HPV16 with HPV18 (any) | 5 | 7 | (2-16) | 2 | 2 | (0-6) | 7 | 4 | (2-8) |
| HPV16 without HPV18 (any) | 34 | 49 | (37-62) | 69 | 57 | (48-66) | 103 | 54 | (47-62) |
| HPV18 without HPV16 (any) | 6 | 9 | (3-18) | 10 | 8 | (4-15) | 16 | 9 | (5-13) |
| HPV16 and/or 18 (without OHR) | 27 | 39 | (28-52) | 40 | 33 | (25-43) | 67 | 35 | (29-43) |
| HPV16 and/or 18 (with OHR) | 18 | 26 | (16-38) | 41 | 34 | (26-43) | 59 | 31 | (25-38) |
| **OHR without HPV 16/18** | **21** | **30** | **(20-43)** | **32** | **27** | **(19-36)** | **53** | **28** | **(22-35)** |
| HR HPV (single infection) | 41 | 59 | (47-71) | 59 | 49 | (40-58) | 100 | 53 | (46-60) |
| **HR HPV (any)** | **66** | **96** | **(88-99)** | **113** | **94** | **(88-98)** | **179** | **95** | **(90-97)** |
| **HPV positive (including LR HPV)** | **66** | **96** | **(88-99)** | **114** | **95** | **(89-98)** | **180** | **95** | **(91-98)** |

HR – high risk; LR – low risk; OHR – Other high risk, excluding HPV 16 or 18; any – regardless of co-infection with other HPV types.

HR HPV includes infection with either type 16, 18, 31, 33, 35, 39, 45, 51, 52, 56, 58, 59 or 68.

P values for differences were not calculated for each specific HPV type between Māori and non-Māori women due to sample size limitations and concerns about multiple comparisons.

Table 3A. Type-specific prevalence of oncogenic HPV infection in women with histologically-confirmed CIN2+

|  | **CIN 2+ (including AIS or glandular dysplasia and cancer) (N=440)** | | | | | | | | |
| --- | --- | --- | --- | --- | --- | --- | --- | --- | --- |
|  | **Māori** | | | **Non-Māori** | | | **Overall** | | |
| **HR HPV type** | **N** | **%** | **95% CI** | **N** | **%** | **95% CI** | **N** | **%** | **95% CI** |
| **HPV16 (any)** | **84** | **55** | **(47-63)** | **153** | **53** | **(47-59)** | **237** | **54** | **(49-59)** |
| HPV52 (any) | 22 | 14 | (9-21) | 55 | 19 | (15-24) | 77 | 17 | (14-21) |
| HPV31 (any) | 19 | 12 | (8-19) | 54 | 19 | (14-24) | 73 | 17 | (13-20) |
| HPV33 (any) | 15 | 10 | (6-16) | 38 | 13 | (10-18) | 53 | 12 | (9-15) |
| HPV18 (any) | 18 | 12 | (7-18) | 34 | 12 | (8-16) | 52 | 12 | (9-15) |
| HPV58 (any) | 16 | 10 | (6-16) | 37 | 13 | (9-17) | 53 | 12 | (9-15) |
| HPV51 (any) | 15 | 10 | (6-16) | 26 | 9 | (6-13) | 41 | 9 | (7-12) |
| HPV39 (any) | 12 | 8 | (4-13) | 19 | 7 | (4-10) | 31 | 7 | (5-10) |
| HPV45 (any) | 6 | 4 | (2-8) | 14 | 5 | (3-8) | 20 | 5 | (3-7) |
| HPV59 (any) | 9 | 6 | (3-11) | 15 | 5 | (3-9) | 24 | 6 | (4-8) |
| HPV35 (any) | 6 | 4 | (2-8) | 11 | 4 | (2-7) | 17 | 4 | (2-6) |
| HPV56 (any) | 8 | 5 | (2-10) | 4 | 1 | (0-4) | 12 | 3 | (1-5) |
| HPV68 (any) | 1 | 1 | (0-4) | 9 | 3 | (1-6) | 10 | 2 | (1-4) |
| **HPV16 and/or 18 (any)** | **96** | **63** | **(55-70)** | **179** | **62** | **(56-68)** | **275** | **63** | **(58-67)** |
| HPV16 with HPV18 (any) | 6 | 4 | (2-8) | 7 | 2 | (1-5) | 13 | 3 | (2-5) |
| HPV16 without HPV18 (any) | 78 | 51 | (43-59) | 146 | 51 | (45-57) | 224 | 51 | (46-56) |
| HPV18 without HPV16 (any) | 12 | 8 | (4-13) | 26 | 9 | (6-13) | 38 | 9 | (6-12) |
| HPV16 and/or 18 (without OHR) | 60 | 39 | (31-47) | 79 | 28 | (22-33) | 139 | 32 | (27-36) |
| HPV16 and/or 18 (with OHR) | 36 | 24 | (17-31) | 100 | 35 | (29-41) | 136 | 31 | (27-35) |
| **OHR without HPV 16/18** | **51** | **33** | **(26-41)** | **94** | **33** | **(27-39)** | **145** | **33** | **(29-38)** |
| HR HPV (single infection) | 89 | 58 | (50-66) | 132 | 46 | (40-52) | 221 | 50 | (45-55) |
| **HR HPV (any)** | **147** | **96** | **(92-99)** | **272** | **95** | **(92-97)** | **419** | **95** | **(93-97)** |
| **HPV positive (including LR HPV)** | **147** | **96** | **(92-99)** | **276** | **96** | **(93-98)** | **423** | **96** | **(94-98)** |

HR – high risk; LR – low risk; OHR – Other high risk, excluding HPV 16 or 18; any – regardless of co-infection with other HPV types.

HR HPV includes infection with either type 16, 18, 31, 33, 35, 39, 45, 51, 52, 56, 58, 59 or 68.

P values for differences were not calculated for each specific HPV type between Māori and non-Māori women due to sample size limitations and concerns about multiple comparisons.

Table 4A. Type-specific prevalence of oncogenic HPV infection in women with histologically-confirmed CIN3+

|  | **CIN 3+ (including AIS or glandular dysplasia and cancer) (N=211)** | | | | | | | | |
| --- | --- | --- | --- | --- | --- | --- | --- | --- | --- |
|  | **Māori** | | | **Non-Māori** | | | **Overall** | | |
| **HR HPV type** | **N** | **%** | **95% CI** | **N** | **%** | **95% CI** | **N** | **%** | **95% CI** |
| **HPV16 (any)** | **42** | **58** | **(45-69)** | **78** | **57** | **(48-65)** | **120** | **57** | **(50-64)** |
| HPV52 (any) | 12 | 16 | (9-27) | 29 | 21 | (15-29) | 41 | 19 | (14-25) |
| HPV31 (any) | 7 | 10 | (4-19) | 18 | 13 | (8-20) | 25 | 12 | (8-17) |
| HPV33 (any) | 5 | 7 | (2-15) | 12 | 9 | (5-15) | 17 | 8 | (5-13) |
| HPV18 (any) | 12 | 16 | (9-27) | 18 | 13 | (8-20) | 30 | 14 | (10-20) |
| HPV58 (any) | 6 | 8 | (3-17) | 14 | 10 | (6-16) | 20 | 10 | (6-14) |
| HPV51 (any) | 8 | 11 | (5-20) | 9 | 7 | (3-12) | 17 | 8 | (5-13) |
| HPV39 (any) | 6 | 8 | (3-17) | 9 | 7 | (3-12) | 15 | 7 | (4-11) |
| HPV45 (any) | 5 | 7 | (2-15) | 8 | 6 | (3-11) | 13 | 6 | (3-10) |
| HPV59 (any) | 4 | 6 | (2-13) | 7 | 5 | (2-10) | 11 | 5 | (3-9) |
| HPV35 (any) | 0 | 0 | (0-5) | 3 | 2 | (0-6) | 3 | 1 | (0-4) |
| HPV56 (any) | 4 | 6 | (2-13) | 1 | 1 | (0-4) | 5 | 2 | (1-5) |
| HPV68 (any) | 0 | 0 | (0-5) | 4 | 3 | (1-7) | 4 | 2 | (1-5) |
| **HPV16 and/or 18 (any)** | **49** | **67** | **(55-78)** | **93** | **67** | **(59-75)** | **142** | **67** | **(61-74)** |
| HPV16 with HPV18 (any) | 5 | 7 | (2-15) | 2 | 1 | (0-5) | 7 | 3 | (1-7) |
| HPV16 without HPV18 (any) | 37 | 51 | (39-63) | 76 | 55 | (46-64) | 113 | 54 | (47-60) |
| HPV18 without HPV16 (any) | 7 | 10 | (4-19) | 15 | 11 | (6-17) | 22 | 10 | (7-15) |
| HPV16 and/or 18 (without OHR) | 31 | 42 | (31-55) | 47 | 34 | (26-43) | 78 | 37 | (30-44) |
| HPV16 and/or 18 (with OHR) | 18 | 25 | (15-36) | 46 | 33 | (26-42) | 64 | 30 | (24-37) |
| **OHR without HPV 16/18** | **21** | **29** | **(19-41)** | **34** | **25** | **(18-33)** | **55** | **26** | **(20-33)** |
| HR HPV (single infection) | 45 | 62 | (50-73) | 68 | 49 | (41-58) | 113 | 54 | (47-60) |
| **HR HPV (any)** | **70** | **96** | **(88-99)** | **127** | **92** | **(86-96)** | **197** | **93** | **(89-96)** |
| **HPV positive (including LR HPV)** | **70** | **96** | **(88-99)** | **129** | **93** | **(88-97)** | **199** | **94** | **(90-97)** |

HR – high risk; LR – low risk; OHR – Other high risk, excluding HPV 16 or 18; any – regardless of co-infection with other HPV types.

HR HPV includes infection with either type 16, 18, 31, 33, 35, 39, 45, 51, 52, 56, 58, 59 or 68.

P values for differences were not calculated for each specific HPV type between Māori and non-Māori women due to sample size limitations and concerns about multiple comparisons.

Table 5A. Age-specific prevalence of grouped HPV types in women with (a) high grade cytology; (b) histologically-confirmed CIN 2+; and (c) histologically-confirmed CIN 3+, including AIS or glandular dysplasia and cancer

|  |  | **ASC-H/HSIL/AGC/AIS cytology (N=730)** | | | | | **CIN 2+ (N=440)** | | | | | **CIN 3+ (N=211)** | | | | |
| --- | --- | --- | --- | --- | --- | --- | --- | --- | --- | --- | --- | --- | --- | --- | --- | --- |
|  |  | **Māori** | | **Non-Māori** | | **X2 test**^b^ | **Māori** | | **Non-Māori** | | **X2 test**^b^ | **Māori** | | **Non-Māori** | | **X2 test**^b^ |
| **HR HPV** | **Age group** | **N^a^** | **% (95% CI)** | **N^a^** | **% (95% CI)** | **P value** | **N^a^** | **% (95% CI)** | **N^a^** | **% (95% CI)** | **P value** | **N^a^** | **% (95% CI)** | **N^a^** | **% (95% CI)** | **P value** |
| HPV 16 (any) | 20-29 | 121 | 52 (43-61) | 214 | 52 (45-59) | 0.97 | 78 | 54 (42-65) | 129 | 62 (53-70) | 0.25 | 35 | 60 (42-76) | 62 | 69 (56-80) | 0.35 |
|  | 30-39 | 77 | 48 (37-60) | 149 | 44 (36-52) | 0.53 | 50 | 54 (39-68) | 101 | 50 (40-61) | 0.69 | 29 | 55 (36-74) | 47 | 53 (38-68) | 0.87 |
|  | 40-69 | 43 | 44 (29-60) | 126 | 25 (18-34) | 0.02 | 25 | 60 (39-79) | 57 | 39 (26-52) | 0.07 | 9 | 56 (21-86) | 29 | 34 (18-54) | 0.26 |
|  | ***Test for trend*** ^c^ | *P=0.39* | | *P=0.001* | | *-* | *P=0.55* | | *P=0.01* | | *-* | *P=0.89* | | *P=0.02* | | *-* |
| HPV 16 and/or | 20-29 | 121 | 60 (50-68) | 214 | 62 (55-68) | 0.50 | 78 | 58 (46-69) | 129 | 71 (62-78) | 0.17 | 35 | 66 (48-81) | 62 | 79 (67-88) | 0.15 |
| 18 (any) | 30-39 | 77 | 58 (47-70) | 149 | 52 (43-60) | 0.52 | 50 | 64 (49-77) | 101 | 59 (49-69) | 0.19 | 29 | 66 (46-82) | 47 | 62 (46-75) | 0.85 |
|  | 40-69 | 43 | 56 (40-71) | 126 | 34 (26-43) | 0.01 | 25 | 76 (55-91) | 57 | 49 (36-63) | 0.05 | 9 | 78 (40-97) | 29 | 52 (33-71) | 0.28 |
|  | ***Test for trend*** ^c^ | *P=0.75* | | *P=0.001* | | *-* | *P=0.07* | | *P=0.03* | | *-* | *P=0.42* | | *P=0.09* | | *-* |
| OHR without | 20-29 | 121 | 36 (28-46) | 214 | 33 (27-40) | 0.39 | 78 | 41 (30-53) | 129 | 28 (20-36) | 0.13 | 35 | 34 (19-52) | 62 | 21 (12-33) | 0.15 |
| HPV 16/18 | 30-39 | 77 | 29 (19-40) | 149 | 35 (27-43) | 0.63 | 50 | 26 (15-40) | 101 | 37 (27-47) | 0.19 | 29 | 24 (10-44) | 47 | 30 (17-45) | 0.85 |
|  | 40-69 | 43 | 37 (23-53) | 126 | 38 (30-47) | 0.02 | 25 | 24 (9-45) | 57 | 37 (24-51) | 0.08 | 9 | 22 (3-60) | 29 | 24 (10-44) | 0.38 |
|  | ***Test for trend*** ^c^ | *P=0.84* | | *P=0.03* | | *-* | *P=0.07* | | *P=0.06* | | *-* | *P=0.42* | | *P=0.29* | | *-* |

HR – high risk; OHR – Other high risk, excluding HPV 16 or 18; any – regardless of co-infection with other HPV types.

HR HPV includes infection with either type 16, 18, 31, 33, 35, 39, 45, 51, 52, 56, 58, 59 or 68.

^a^ Total number of women at each age group by ethnicity (i.e. including women HPV negative).

^b^ Chi-square test for difference between Māori and non-Māori women in the age-specific prevalence of grouped HPV types.

^c^ Test for trend across each level of age group.

Table 6A. Likelihood of having grouped HPV infection in HPV positive women with (a) high grade cytology; (b) histologically-confirmed CIN 2+ (including cancer); and (c) histologically-confirmed CIN 3+ (including cancer)

| **Category** | **Any HPV 16 positive^a^** | | | **Any HPV 16 and/or 18 positive^a^** | | | **Other HR HPV without HPV 16 and/or 18** | | |
| --- | --- | --- | --- | --- | --- | --- | --- | --- | --- |
|  | **ASC-H/HSIL cytology**  **OR (95% CI)** | **CIN 2+**  **OR (95% CI)** | **CIN 3+**  **OR (95% CI)** | **ASC-H/HSIL cytology**  **OR (95% CI)** | **CIN 2+**  **OR (95% CI)** | **CIN 3+**  **OR (95% CI)** | **ASC-H/HSIL cytology**  **OR (95% CI)** | **CIN 2+**  **OR (95% CI)** | **CIN 3+**  **OR (95% CI)** |
| Non-Māori, 20-29 years | 1.0 | 1.0 | 1.0 | 1.0 | 1.0 | 1.0 | 1.0 | 1.0 | 1.0 |
| Non-Māori, 30-39 years | 0.7 (0.5-1.1) | 0.6 (0.4-1.1) | 0.5 (0.2-1.1) | 0.8 (0.5-1.2) | 0.7 (0.4-1.2) | 0.6 (0.2-1.3) | 1.2 (0.8-1.9) | 1.6 (0.9-2.8) | 1.8 (0.8-4.4) |
| Non-Māori, 40-69 years | 0.3 (0.2-0.5) | 0.4 (0.2-0.7) | 0.2 (0.1-0.6) | 0.4 (0.3-0.7) | 0.5 (0.3-1.0) | 0.4 (0.2-1.2) | 1.8 (1.1-2.8) | 1.8 (0.9-3.5) | 1.6 (0.5-4.5) |
| Māori, 20-29 years | 1.0 (0.6-1.6) | 0.7 (0.4-1.3) | 0.7 (0.3-1.6) | 1.0 (0.6-1.5) | 0.6 (0.3-1.0) | 0.5 (0.2-1.3) | 1.2 (0.7-1.9) | 1.8 (1.0-3.3) | 2.0 (0.8-5.0) |
| Māori, 30-39 years | 0.9 (0.5-1.4) | 0.7 (0.4-1.4) | 0.5 (0.2-1.4) | 1.1 (0.6-1.9) | 1.0 (0.5-2.1) | 0.7 (0.2-2.1) | 0.9 (0.5-1.6) | 1.0 (0.5-2.2) | 1.4 (0.5-4.0) |
| Māori, 40-69 years | 0.7 (0.4-1.4) | 0.9 (0.4-2.2) | 0.6 (0.1-2.3) | 0.8 (0.4-1.6) | 1.3 (0.5-3.5) | 0.9 (0.2-5.0) | 1.2 (0.6-2.4) | 0.8 (0.3-2.2) | 1.1 (0.2-5.8) |
| *Test for interaction^b^* | *P=0.15* | *P=0.1* | *P=0.35* | *P=0.29* | *P=0.02* | *P=0.25* | *P=0.34* | *P=0.03* | *P=0.25* |

Other HR HPV types include infection with either type 31, 33, 35, 39, 45, 51, 52, 56, 58, 59 or 68, not 16 or 18.

^a^ Regardless of co-infection with other HPV types.

^b^ Logistic regression including an interaction term for ethnicity and age group
